# Aluminium-induced reduction of plant growth in alfalfa (*Medicago sativa*) is mediated by interrupting auxin transport and accumulation in roots

**DOI:** 10.1038/srep30079

**Published:** 2016-07-20

**Authors:** Shengyin Wang, Xiaoyan Ren, Bingru Huang, Ge Wang, Peng Zhou, Yuan An

**Affiliations:** 1 School of Agriculture and Biology, Shanghai Jiao Tong University, Shanghai 200240, P. R. China; 2 Department of Plant Biology and Pathology, Rutgers, The State University of New Jersey, New Jersey, NJ 08901, USA; 3 Instrumental Analysis Centre of Shanghai Jiao Tong University, Shanghai Jiao Tong University, Shanghai 200240, P. R. China; 4 Key Laboratory of Urban Agriculture (South), Ministry of Agriculture, Shanghai 201101, China

## Abstract

The objective of this study was to investigate Al^3+^-induced IAA transport, distribution, and the relation of these two processes to Al^3+^-inhibition of root growth in alfalfa. Alfalfa seedlings with or without apical buds were exposed to 0 or 100 μM AlCl_3_ and were foliar sprayed with water or 6 mg L^−1^ IAA. Aluminium stress resulted in disordered arrangement of cells, deformed cell shapes, altered cell structure, and a shorter length of the meristematic zone in root tips. Aluminium stress significantly decreased the IAA concentration in apical buds and root tips. The distribution of IAA fluorescence signals in root tips was disturbed, and the IAA transportation from shoot base to root tip was inhibited. The highest intensity of fluorescence signals was detected in the apical meristematic zone. Exogenous application of IAA markedly alleviated the Al^3+^-induced inhibition of root growth by increasing IAA accumulation and recovering the damaged cell structure in root tips. In addition, Al^3+^ stress up-regulated expression of *AUX1* and *PIN2* genes. These results indicate that Al^3+^-induced reduction of root growth could be associated with the inhibitions of IAA synthesis in apical buds and IAA transportation in roots, as well as the imbalance of IAA distribution in root tips.

Aluminium (Al) is the most abundant metal and is widely distributed in nature in the form of silicates or other deposits^1^. A high content of soluble Al^3+^ in soils with a pH below 5 is very phytotoxic and becomes a major limiting factor of plant productivity in acidic soils^2^. The most obvious symptom of Al^3+^ toxicity is the inhibition of root growth^3^. Excessive Al^3+^ inhibits both cell elongation and division in roots, leading to swollen root apices with poor or no root-hair development. Several studies suggested that this may be caused by the interaction of Al^3+^ with signal transduction pathways regulating cell growth. It has been shown that Al^3+^ may be bound to the plasma membrane, disturbing ion transport and homeostasis as well as blocking Ca^2+^-dependent signalling cascades^4^. The binding of Al^3+^ to the plasma membrane may also lead to a disruption of membrane function and the promotion of oxidative stress, which prevent the release of secondary signalling molecules and influence the organization of the cell cytoskeleton^5^,^6^. In addition, Al^3+^ stress induces callose production in plasmodesmata, which may block auxin or indole acetic acid (IAA) transport through the symplast and result in a Al^3+^-induced inhibition of root cell elongation^7^,^8^.

Plant hormones are involved in plant adaptation to environmental stresses, including Al^3+^ stress^9^,^10^,^11^. The involvement of auxin in a plant’s response to heavy metal toxicity has been investigated in recent years. For example, cadmium toxicity altered IAA distribution by increasing the activity of IAA oxidase in roots of *Medicago truncatula* seedlings^12^ and disturbed auxin homeostasis by affecting the distribution, metabolism, and transport of auxin in *Arabidopsis* seedlings^13^,^14^. Additionally, auxin transport via AUXIN RESISTANT 1 (AUX1) has been shown to play a positive role in plant tolerance to arsenite stress by influencing reactive oxygen species (ROS)-mediated signalling pathways^15^. Finally, copper (Cu^2+^) treatments modulated auxin redistribution via regulation of PINFORMED1 (PIN1) in *Arabidopsis* roots, and exogenous application of auxin reduced the toxic effect of Cu^2+^ on sunflower (*Helianthus annuus*), leading to an increase in root length and root hair formation^16^,^17^. However, limited information is available on the roles that auxin plays in plant responses to Al^3+^ stress.

Alfalfa (*Medicago sativa* L.) is an important legume and is used as a forage crop worldwide, but aluminium toxicity is a major factor limiting alfalfa production in soils with low pH^18^,^19^. Understanding the mechanisms underlying IAA regulation of alfalfa’s response to Al^3+^ is important for developing Al^3+^-tolerant germplasm through genetic modification or molecular breeding. Our previous study showed that IAA concentrations in apical buds and root tips of Al^3+^-stressed seedlings decreased in a short period (1–3 d) of Al^3+^ stress^20^, which may reduce the growth of alfalfa seedlings under Al^3+^ stress. In the present study, we aimed to further investigate the following questions: (1) What was the reason of IAA accumulation decrease in the Al^3+^-stressed root tips? (2) What was the effect of Al^3+^ stress on cell structure and IAA distribution in root tips? (3) Was there any effect of IAA on the cell structure formation of the Al^3+^-stressed root tips?

## Results

### IAA alleviated Al^3+^-induced plant growth

**Figure 1:**
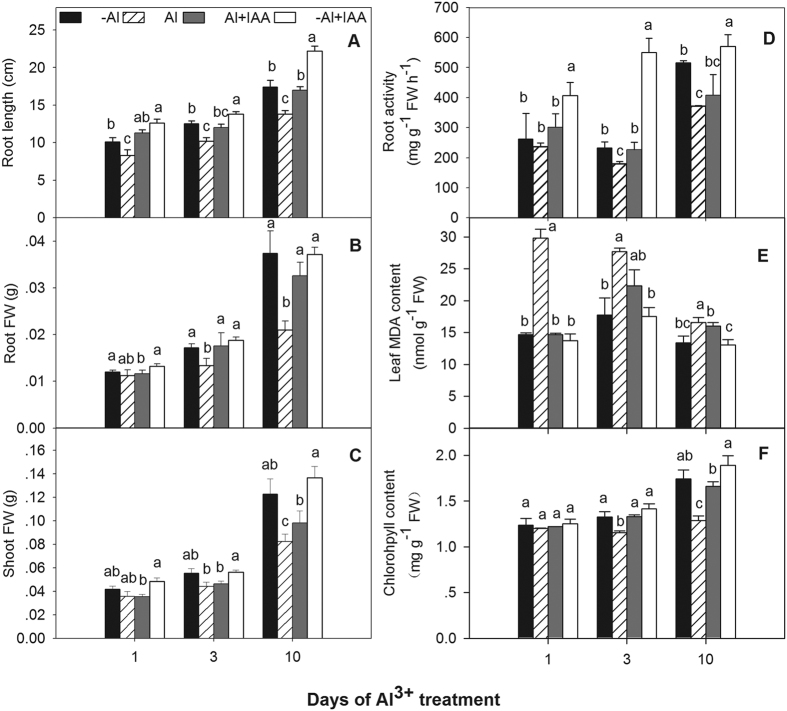
Root length (**A**), shoot (**B**), root (**C**) fresh weight (FW), root activity (**D**), leaf MDA content (**E**), and total chlorophyll content (**F**) in alfalfa seedlings grown for 1, 3 and 10 days on ½-strength Hoagland’s medium (pH 4.5) with treatments of no aluminium (-Al), aluminium stress (Al) (100 μM AlCl_3_), aluminium with additional IAA (Al + IAA) (100 μM AlCl_3_ + IAA) and -Al with additional IAA (-Al + IAA). Data are means ± SE of three replicates. Bars with different letters indicate a significant difference at *P* < 0.05 (least significant difference test).

The primary Al^3+^ toxicity in plants is the inhibition of root growth. When alfalfa seedlings were exposed to varying concentrations of AlCl_3_ (0, 50, 100, 200 or 300 μM) for 48 h, the inhibition of roots elongation was positively dependent on AlCl_3_ concentrations, in which the higher decline rate of relative root growth was in the Al^3+^ concentration of 100 μM (Supplemental Fig. 1). Root length of alfalfa exposed to 100 μM AlCl_3_ was significantly reduced by 17–21% relative to plants without Al^3+^ treatments (-Al, control) from 1 to 10 d of treatment (Fig. 1A). Root fresh weight (Fig. 1B), shoot fresh weight (Fig. 1C), root activity (Fig. 1D) and total chlorophyll content (Fig. 1F) were significantly decreased after 3 d of Al^3+^ treatment and were 56.1, 32.9, 27.9 and 26.2% lower than those in control plants at 10 d of Al^3+^ treatment, respectively. The MDA content in leaves of Al^3+^-stressed plants (Fig. 1E) was significantly increased compared to control plants during the experimental periods.

Compared with Al^3+^ treatment alone, the addition of IAA (Al + IAA) markedly increased root length (Fig. 1A), root fresh weight (Fig. 1B), shoot fresh weight (Fig. 1C), and total chlorophyll content (Fig. 1F) after 10 d of the experiment. The MDA content in leaves treated with Al + IAA decreased to levels near those of the control plants (-Al) (Fig. 1E).

### IAA alleviated Al^3+^-induced damage of root tip structure

**Figure 2:**
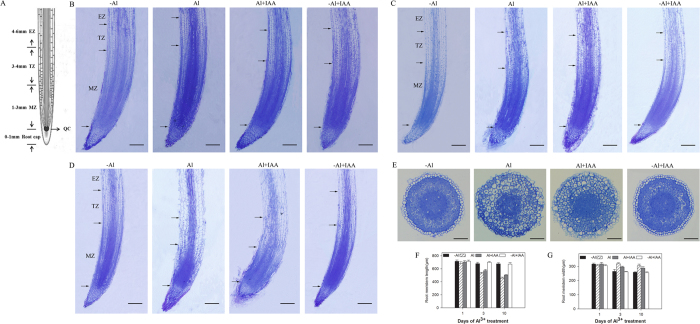
Images of root tips (0–6 mm) in the middle longitudinal section of alfalfa seedlings grown for 1, 3 and 10 days on ½-strength Hoagland’s medium (pH 4.5) with treatments of no aluminium (-Al), aluminium stress (Al) (100 μM AlCl_3_), aluminium with additional IAA (Al + IAA) (100 μM AlCl_3_ + IAA) and -Al with additional IAA (-Al + IAA). Root tip (**A**) was divided into the root cap, the transition zone (TZ), the meristematic zone (MZ) and the elongation zone (EZ) and is indicated by arrowheads. The quiescent centre was marked as QC. A cross-section was cut in the middle of the meristematic zone (**E**) on day 10. Scale bars in longitudinal sections indicate 100 μm; in cross-sections, they indicate 50 μm. The length (**F**) and width (**G**) of the root meristems were measured. Data are means ± SE of three replicates. Bars with different letters indicate a significant difference at *P* < 0.05 (least significant difference test).

Root tips mainly contain four regions: the root cap (0–1 mm from the tip), meristematic zone (MZ 1–3 mm), transition zone (TZ 3–4 mm) and elongation zone (EZ 4–6 mm) (Fig. 2A). Compared with the control, Al^3+^ treatments decreased the root meristem length at 1, 3 and 10 d (Fig. 2B–D,F) and enhanced the root meristem width at 3 and 10 d (Fig. 2G). The tissue structure in the whole root tip was damaged by the addition of Al^3+^. The cell arrangement was also disordered, and the cell shape was distorted from a rectangle into an irregular shape in the epidermis and cortex and even in the stele of the TZ and EZ (Fig. 2C,D). The cell length enlarged in the cortex in all root tip zones, the epidermal cells in the root cap detached, and the root tips became obtuse (Fig. 2D). Compared with the control (-Al) and based on the cross-section in the middle of the MZ (Fig. 2E), the epidermal cell was seriously damaged, the cell and vacuole enlarged, the intercellular space diminished, the thickness of the cortex increased, and the diameter of the stele decreased. However, the addition of IAA (Al + IAA) protected the tissue structure of the Al^3+^-stressed root tip in which the cell arrangement, cell shape and cell length as well as the thickness of the cortex were close to those of the control plant (no Al^3+^ addition), and the meristem length increased compared with Al^3+^ treatment alone (Fig. 2A–F).

### Al^3+^ altered IAA distribution in plants

**Figure 3:**
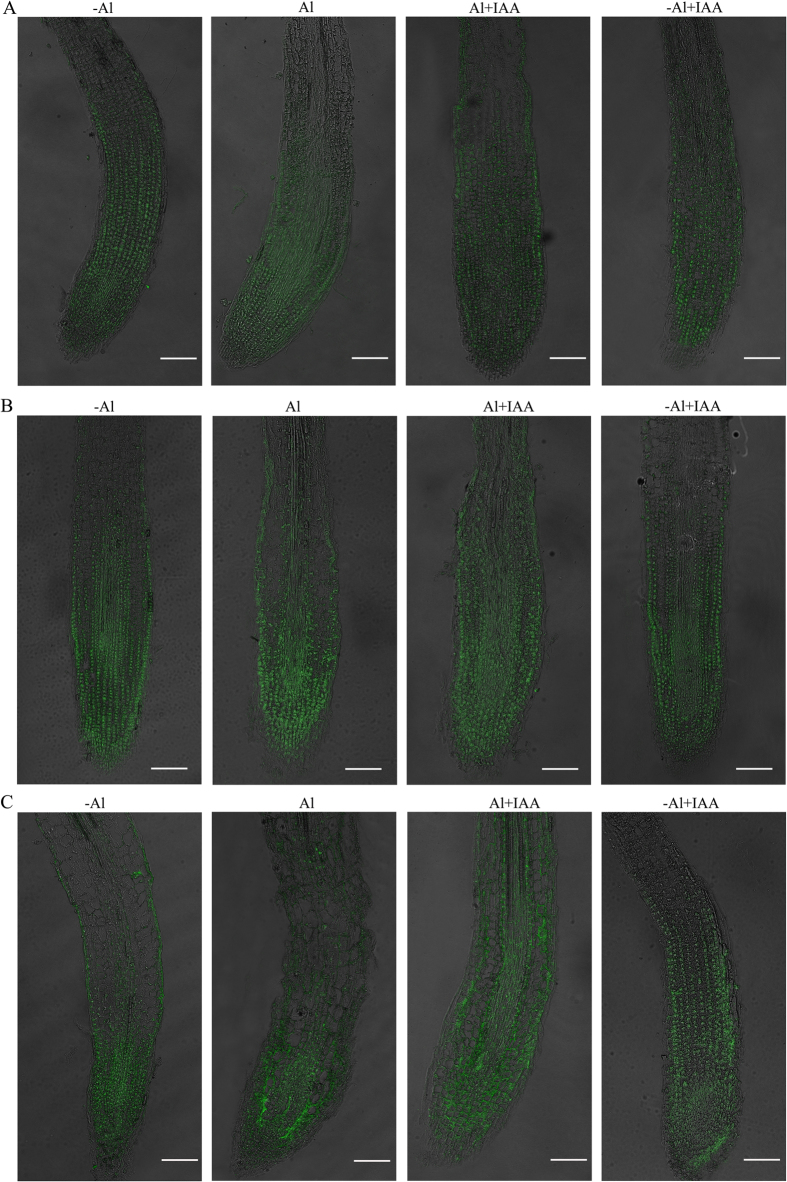
Immunolocalization of IAA in the middle longitudinal section of alfalfa seedlings grown for 1, 3 and 10 days on ½-strength Hoagland’s medium (pH 4.5) with treatments of no aluminium (-Al), aluminium stress (Al) (100 μM AlCl_3_), aluminium with additional IAA (Al + IAA) (100 μM AlCl_3_ + IAA) and -Al with additional IAA (-Al + IAA). Scale bars in each image indicate 100 μm.

To explore the IAA distribution in alfalfa root tips, the immunohistochemical method of staining with antibodies was used in this study. In the control (-Al) and -Al + IAA treatments, a high intensity of homogeneous fluorescence signals was detected as a continuous dotted line in the epidermis, cortex (close to the endodermis), endodermis and stele of the TZ and distal MZ (Fig. 3A,B), whereas weak signals was detected in the EZ during the 10-d experimental period (Fig. 3C). However, in the Al^3+^ treatment alone, the distribution of fluorescence signals in root tips was disturbed; the dotted line characterizing fluorescence signals disappeared (Fig. 3B,C). Discontinuous fluorescence signals were detected in the epidermis, cortex (close to the endodermis), endodermis, and stele of the TZ and distal MZ. On the other hand, a higher intensity of fluorescence signals was detected in all tissues of the apical MZ compared with the control (-Al) treatment after 10 d of Al^3+^ treatment (Fig. 3B,C), which means that the distribution of IAA was altered in root tips. Higher IAA accumulation in the apical MZ and lower IAA accumulation in the TZ compared with the control (-Al) treatment indicated that IAA transportation in root tips was inhibited.

In the combined treatment of Al^3+^ with IAA (Al + IAA), the IAA signals were less disturbed by Al^3+^ stress (Fig. 3A–C); the dotted lines of fluorescence signals were clearer and more continuous than in Al^3+^ treatment alone after 3 d of Al^3+^ treatment (Fig. 3A,B). The distributional intensity of fluorescence signals in the whole root tip was also close to that of the control plant. The IAA signals in the epidermis, cortex and stele of the TZ were increased compared with the Al^3+^ (Al) treatment, indicating that exogenous application of IAA could compensate for the lack of IAA in the TZ under Al^3+^ (Al) treatment. Either the primary antibody or the secondary antibody was omitted during the complete procedure, and no signals were observed in the two antibody breeding procedures (Supplementary Fig. 2).

**Figure 4:**
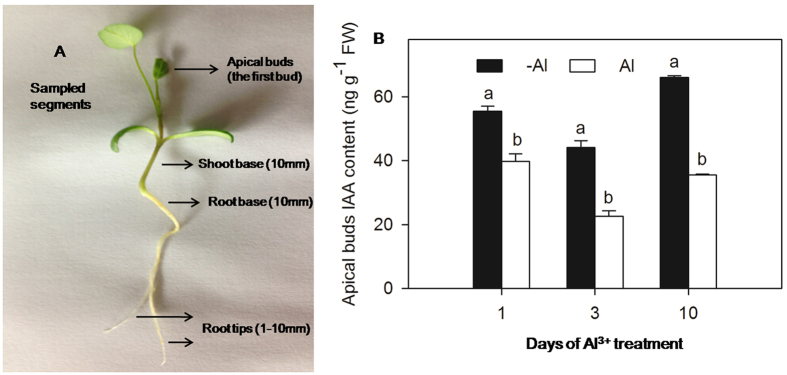
Sampled segments are shown in (**A**); IAA concentrations in apical buds of alfalfa seedlings grown for 1, 3 and 10 days on ½-strength Hoagland’s medium (pH 4.5) in the treatments of no aluminium (-Al) and aluminium stress (Al) (100 μM AlCl_3_) (**B**). Data are means ± SE of three replicates. Bars with different letters indicate a significant difference at *P* < 0.05 (least significant difference test).

To evaluate the mechanism by which exogenous application of IAA alleviates Al^3+^ toxicity, IAA concentration in different parts of the plant was measured. Sampled segments are shown in Fig. 4A. When alfalfa was exposed to 100 μM AlCl_3_, IAA concentrations in apical buds (Fig. 4B) and root tips at 1, 3, and 10 d as well as in the shoot base at 3 and 10 d were significantly reduced compared to control plants (Table 1). However, the degree of reduction in apical buds (25–50%) was lower than in root tips (75–90%) (Table 1). IAA concentrations in the root base were significantly higher in both the Al^3+^ treatment and Al^3+^ with IAA treatment (Al + IAA) compared to both the treatment without Al^3+^ (-Al) and -Al^3+^ with IAA treatment (-Al + IAA), respectively, after 3 d (Table 1).

Compared to Al^3+^ treatment alone, Al + IAA significantly increased (by 40–140%) IAA concentrations in the shoot base throughout the experimental period; however, the increased IAA concentration in root tips reached a significant level only at 1 d of Al + IAA treatment (Table 1). In contrast to the treatment without Al^3+^ (-Al), -Al + IAA induced an increase in IAA concentrations in both the shoot base and root tips, by 20–88% and 25–96%, respectively, throughout the experimental period (Table 1). After exogenous application of IAA, the degree of increase in IAA in root tips was lower in the plants treated with Al^3+^ than in those without Al^3+^. In addition, regardless of IAA treatment, the ratio of IAA concentrations between root tips and shoot base was lower in the presence of Al^3+^ than without Al^3+^ throughout the experimental period (Table 1).

**Figure 5:**
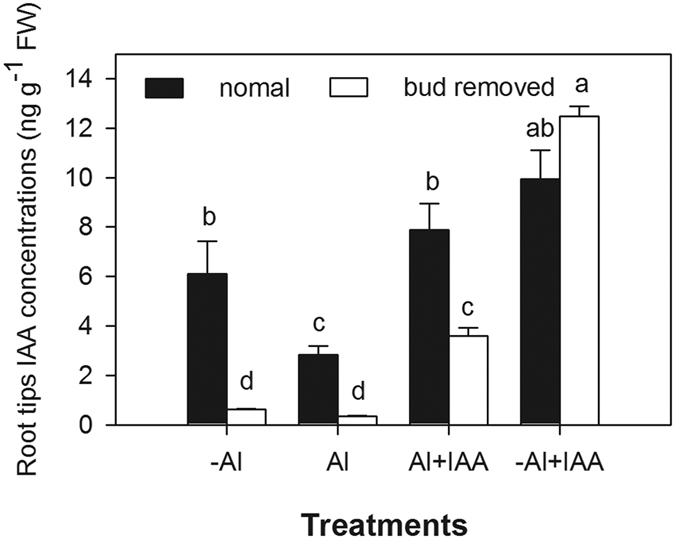
IAA concentrations in root tips (0–10 mm) of alfalfa seedlings (normal or apical bud removed) grown for 24 h on ½-strength Hoagland’s medium (pH 4.5) with treatments of no aluminium (-Al), aluminium stress (Al) (100 μM AlCl_3_), aluminium with additional IAA (Al+IAA) (100 μM AlCl_3_ + IAA) and -Al with additional IAA (-Al + IAA). Data are means ± SE of three replicates. Bars with different letters indicate a significant difference at *P* < 0.05 (least significant difference test).

To further explore the effect of Al^3+^ on IAA distribution in the plant, apical buds that were a primary site for IAA synthesis were removed 24 h before Al^3+^ treatment; this was done to eliminate the effect of endogenous IAA synthesis. Compared with plants with intact apical buds, the IAA concentration in root tips was significantly decreased in plants without apical buds in both the -Al and Al treatment, by 89.8% and 87.6%, respectively (Fig. 5). In bud-removed plants, exogenous application of IAA significantly increased IAA concentrations in root tips in both -Al + IAA and Al + IAA treatments, 25-fold and 10-fold higher than those in -Al^3+^ and Al^3+^ treatments, respectively (Fig. 5). The degree of increase was lower in the Al^3+^ treatment than in the treatment without Al^3+^.

### Magnesium (Mg^2+^) and zinc (Zn^2+^) alleviated Al^3+^-induced inhibition of root elongation by increased IAA accumulation in root tips

**Figure 6:**
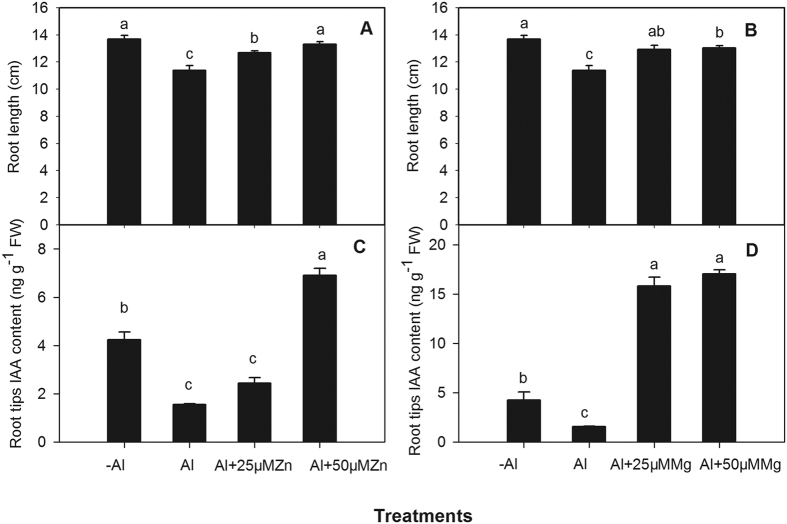
Root length and IAA concentrations in root tips (0–10 mm) of alfalfa seedlings grown for 24 h on ½-strength Hoagland’s medium (pH 4.5) with treatments of no aluminium (-Al), aluminium stress (Al) (100 μM AlCl_3_), aluminium together with 25 μM Zn^2+^ (Al + 25 μM Zn), aluminium together with 50 μM Zn^2+^ (Al + 50 μM Zn) (**A,C**), aluminium together with 25 μM Mg^2+^ (Al + 25 μM Mg) and aluminium together with 50 μM Mg^2+^ (Al + 50 μM Mg)(**B,D**). Data are means ± SE of three replicates. Bars with different letters indicate a significant difference at *P* < 0.05 (least significant difference test).

Compared with Al^3+^ treatment alone, the addition of Mg^2+^ (Fig. 6A) and Zn^2+^ (Fig. 6B) to the Al^3+^ treatment significantly increased root length, which reached the same level as the control (-Al) after 1 d of treatment. The addition of 25 μM and 50 μM Mg^2+^ with excess Al^3+^ for 1 d also significantly increased IAA concentrations in the root tips 9.2-fold and 9.5-fold, respectively, in comparison with Al^3+^ treatment (Fig. 6C). The addition of 25 μM or 50 μM Zn^2+^ significantly increased IAA concentrations in the root tips by 56.73 and 340%, respectively (Fig. 6D).

### IAA decreased Al^3+^-induced callose production in plants

**Figure 7:**
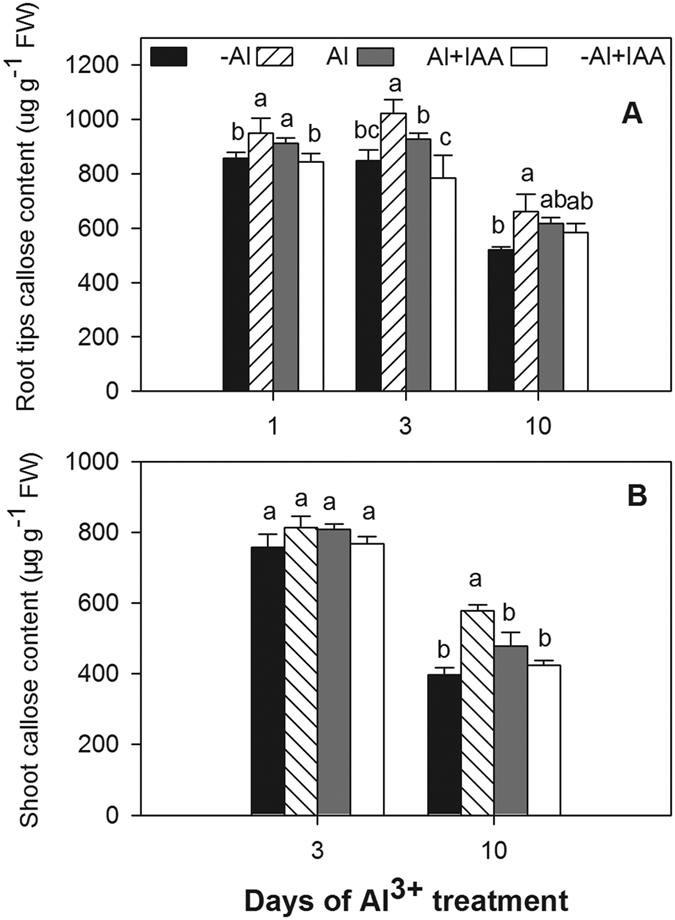
Callose content in root tips (0–10 mm) (**A**) and shoot (**B**) of alfalfa seedlings grown for 1, 3 and 10 days on ½-strength Hoagland’s medium (pH 4.5) with treatments of no aluminium (-Al), aluminium stress (Al) (100 μM AlCl_3_), aluminium with additional IAA (Al + IAA) (100 μM AlCl_3_ + IAA) and -Al with additional IAA (-Al + IAA). Data are means ± SE of three replicates. Bars with different letters indicate a significant difference at *P* < 0.05 (least significant difference test).

Compared to the treatment without Al^3+^ (-Al), callose content in root tips increased by 10.9, 20.4 and 27.0% at 1, 3 and 10 d of Al^3+^ treatment, respectively (Fig. 7A). Callose content in shoots under Al^3+^ treatment also increased by 45.9% at 10 d but showed no significant difference from the content under -Al treatment at 3 d (Fig. 7B). This finding suggests that shoot callose formation was slower than root callose formation under Al^3+^ stress. Compared to Al^3+^ treatment, callose content reduction was only 9.1% in root tips and 17.2% in shoots in the Al + IAA treatment throughout the experimental period (Fig. 7).

### Al^3+^ up-regulated expression levels of IAA transport related genes

**Figure 8:**
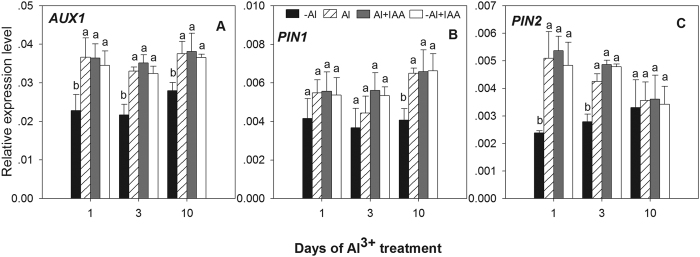
Expression of *AUX1, PIN1* and *PIN2* in root tips (0–10 mm) (**A–C**) of alfalfa seedlings grown for 1, 3 and 10 days on ½-strength Hoagland’s medium (pH 4.5) in the treatments of no aluminium (-Al) and aluminium stress (Al) (100 μM AlCl_3_) aluminium with additional IAA (Al + IAA) (100 μM AlCl_3_ + IAA) and -Al with additional IAA (-Al + IAA). Data are means ± SE of three replicates. Bars with different letters indicate a significant difference at *P* < 0.05 (least significant difference test).

The relative transcription levels of *AUX1* in root tips were significantly up-regulated by Al^3+^ treatment by 60.5, 52.1 and 30.1% at 1, 3, and 10 d of treatment, respectively (Fig. 8A). The relative expression levels of *PIN2* in Al^3+^ treatment were also significantly up-regulated by 114.1, 52.3 and 7.7% at 1, 3, and 10 d, respectively (Fig. 8C), whereas the expression levels of *PIN1* did not show a significant increase within the first 3 d but exhibited a significant increase at 10 d (Fig. 8B). The relative transcription levels of the three genes were all up-regulated after exogenous application of IAA in both -Al + IAA and Al + IAA treatments compared with -Al treatment, but there was no significant difference in the expression level of the three genes among the treatments of -Al + IAA, Al + IAA and Al (Fig. 8A–C).

## Discussion

### Al^3+^ inhibition of IAA synthesis in apical buds

Various environmental and endogenous signals can be integrated into changes of auxin distribution through their effects on local auxin biosynthesis^10^,^21^. When alfalfa was exposed to Al^3+^-stressed conditions, IAA concentrations in apical buds and root tips were significantly decreased; the IAA concentrations in root tips in both -Al and Al treatments decreased to a very low level after the apical buds were removed. These results indicate that the synthesis of IAA is mainly in apical buds and strongly decreased by Al^3+^ stress, which leads to the reduction of IAA concentration in root tips.

Mg^2+^ is an important cofactor of phenylpyruvate decarboxylase, which is involved in the biosynthesis of IAA^22^. Zn^2+^ is necessary for the formation of IAA from tryptophan, one of the essential ingredients for IAA synthesis, and its concentrations indirectly affect the IAA content in plants^23^. The two nutrients are also extremely important for maintaining membrane function, cell extension and antioxidant enzyme activities^24^. Al^3+^ stress interfered with uptake or transport of nutrients and caused a decrease of Mg content in plant roots of maize (*Zea mays*), rice (*Oryza.sativa*), wheat *(Triticum aestivum*) and alfalfa^6^,^24^,^25^. Exogenous application of Mg^2+^ in culture medium could alleviate Al^3+^ toxicity in many plant species^26^; meanwhile, up-regulation of Mg^2+^ transporter genes, particularly those for high-affinity Mg^2+^ transporters, could contribute to amelioration of Al^3+^ toxicity by enhancing Mg^2+^ uptake in *Arabidopsis*^27^. The proposed mechanisms for Al^3+^ toxicity alleviation by Mg^2+^ ions include (i) increase of organic acid synthesis and exudation; (ii) reduction in Al^3+^ saturation at the apoplastic exchange sites; and (iii) decrease of Al^3+^ activity at the root cell plasma membrane surface, and restored plasma membrane H^+^-ATPase activity^26^,^28^. In present study, the absence of Mg^2+^ and Zn^2+^ resulted in a significant decrease in IAA concentration in root tips of Al^3+^-stressed alfalfa. On the other hand, application of Mg^2+^ and Zn^2+^ significantly increased IAA concentrations in root tips and also enhanced root growth, indicating that Al^3+^-induced deficiency of Mg^2+^ and Zn^2+^ in roots could be associated with the reduction of IAA synthesis in apical buds. The effect of Mg^2+^ on alleviating Al^3+^-induced IAA reduction in root tip was higher than Zn^2+^, The IAA concentrations in root tips under Al^3+^ stress was 6.5-fold and 2.5-fold higher in treatments of 25 μM and 50 μM Mg^2+^ in comparison with treatments of 25 μM and 50 μM Zn^2+^, respectively. The function of the Mg^2+^ in increasing the IAA concentration in Al^3+^-stressed root tip may associate with increase^22^ of IAA biosynthesis and decrease of Al^3+^ activity on the root cell plasma membrane surface reflected by a normal cell structure and a convenient array of IAA fluorescence signals in Al^3+^-stressed root tips after exogenous application IAA.

### Al^3+^ inhibition of IAA transport and disruption of IAA distribution in root tip

IAA is transported from auxin-synthesized young shoot tissues via phloem to the root tip and is then redirected to the epidermis and outer cortex^10^,^29^. Inhibition of basipetal auxin flow disturbs auxin distribution in plants and causes adverse effects on root growth and morphology^10^. In the present study, the ratio of IAA concentration between root tip and shoot base was 2–6 times lower in the presence Al^3+^ than in the absence of Al^3+^; meanwhile, after exogenous application of IAA to plants with or without apical buds, the increased degrees of IAA accumulation in root tips were lower in the plants treated with Al^3+^ than in those with no Al^3+^. In addition, the distribution of fluorescence signals in root tips of Al^3+^-stressed alfalfa was disturbed; the dotted lines of fluorescence signals were not clear and were discontinuous in the endodermis, the cortex (close to the endodermis) and the stele of the TZ and distal MZ. These two results and the phenomenon overall strongly demonstrated that Al^3+^ inhibited IAA transport from shoots to root tips. As a result, a higher amount of IAA accumulated in the root base, but a lower amount accumulated in root tips.

Although Al^3+^ stress caused a reduction of IAA concentration in the root tip, stronger IAA fluorescence signals were presented in the apical MZ, including the quiescent centre (QC), a highly efficient operation centre of IAA polar transport^30^,^31^. As well as fewer IAA signals were observed in the TZ and EZ under Al^3+^ treatment. Using the isotopic tracer method, Kollmeier *et al*.^7^ found that Al^3+^ treatment increased auxin accumulation in the MZ and DTZ (the distal transition zone) but reduced auxin levels in the EZ of maize root tips. These results indicated that Al^3+^ stress decreased IAA accumulation in root tips, but the IAA distribution was unbalanced. The meristematic, transition and elongation zones determined primary root development and root growth. Appropriate concentrations of auxin in the distinct zones could promote cell division and root elongation; for example, an external supply of auxin to the EZ was able to partially overcome the inhibition of root growth in maize caused by Al^3+^ stress^7^, but excessive auxin will reduce cell division and root elongation. Over-accumulation of IAA in the apical meristem might have a negative effect on cell division and root elongation. The reduction and imbalance of IAA accumulation in root tips of Al^3+^-stressed alfalfa could together influence cell division, elongation and differentiation in the distinct zones of roots; as a result, restricted root development and growth.

### Al^3+^-induced cellular response in root tips inhibited IAA transport and root growth

High concentrations of Al^3+^ ions inhibited root development at the organ, tissue, and cellular levels^32^. The Al^3+^ ions were located in the epidermis and stele of Al^3+^-stressed roots^20^, which could inhibit cell division and elongation^33^,^34^, changed cell patterns^35^ in the epidermis and stele, and even damaged the epidermis and stele^36^. An obvious characteristic of root development under Al^3+^-stressed conditions was swollen and short root tips, including fibrous roots. Changes in both cell division and cell elongation could alter root tip morphology. In the present study, the diameter of the stele was decreased, while the cortex width was enlarged due to cell enlargements under Al^3+^ stress, which directly caused swelling of the root tip. The cell enlargement was attributed to the Al^3+^-induced vacuole enlargements. Vacuole enlargement might be necessary for plants to store excessive Al^3+^ and adapt to conditions of Al^3+^ stress^37^,^38^, but this change might disturb root functions such as nutrient absorption, and reduced root growth; thus, vacuole enlargement might be an important characteristic of plant Al^3+^ toxicity.

In addition to the change in cross-section structure of cells, the longitudinal cell arrangement was disordered, the cell shapes were distorted from a rectangle into an irregular shape, and the cell size was enlarged in the cortex and even in the stele of the TZ and EZ. The cell structure changes in the longitudinal direction coincided with the distribution of IAA fluorescence signal intensities in the longitudinal direction of the cortex and stele; in which, the dotted lines of IAA fluorescence signals were discontinuous and disordered. These longitudinal changes in cell structure might inhibit IAA down-transport from the upper root to root tips through the stele, as well as backflow through the epidermis and outer cortex cells and lead to an imbalance in IAA accumulation in root tips. Meanwhile, Al^3+^ stress promoted differentiation of the root meristematic zone and shorted the length of the MZ, which led to an early appearance of the TZ and EZ, as well as the cells in the TZ and EZ were seriously enlarged. Doncheva *et al*.^39^ reported that Al^3+^ ions could inhibit cell division in the proximal meristem (250–800 μm from the tip), but stimulated cell division in the elongation zone of the root tip. Thus, the meristematic zone was very sensitive to Al^3+^, and the cell division inhibition in MZ directly reduced the amount of new cell supplement in roots, as a consequence, fundamentally restricted root elongation.

Callose (β-1,3 binding glucan), a sensitive marker of Al^3+^ sensitivity, was quickly induced in the epidermis and cortex in Al^3+^-stressed plants. It was deposited along the plasmodesmata sleeve and blocked symplastic transport and communication^5^,^36^,^40^. The Al-induced callose accumulation in shoots and root tips blocked auxin movement down the stele to the root tips as well as the backward flow to the upper root in the epidermis and outer cortex cell, leading to a disturbed IAA distribution in root tips.

The accumulation of auxin in the root tip is mediated by its biosynthesis and transport^10^,^29^. Polar auxin transport is the central rate-limiting component of auxin transport^29^,^41^. Polar auxin transport in the root epidermis requires PIN2 and AUX1^41^,^42^. PIN2 plays a pivotal role in mediating the backward (towards the root base) auxin flow in the epidermis and outer cortex of cells^43^. The *AUX1* gene encodes a transmembrane protein and is believed to be associated with the influx of auxin across the plasma membrane^42^. Transcriptional analysis demonstrated that *PIN2* and *AUX1* were up-regulated in root tips exposed to Al^3+^, positively mediating polar auxin transport from the QC of root tips to the upper root, leading to a higher IAA accumulation in the epidermis and cortex of the meristematic zone; this was reflected by higher intensity of IAA fluorescence signals. Because the cell structure and arrangement in the cortex of the TZ and EZ were changed, and aluminum-induced cell change would alter cell cytoskeleton^8^, the auxin transport within both cells and inter-cellularly might be affected^35^,^44^. Therefore, the IAA fluorescence signals discontinued in the cortex in the TZ and the backward IAA transport was blocked, leading to a higher auxin accumulation in the meristematic zone.

*PIN1* is another gene functioning in polar auxin transport and helps auxin flow from apical buds to the root tips through the stele^10^. Transcription of this gene was up-regulated in Al^3+^-stressed roots in the present study. This finding suggests that the PIN1 regulation of polar auxin transport through the stele was not affected by Al^3+^ stress in alfalfa and that the reduction of IAA transport through the stele to root tips may be regulated by other mechanisms in which the Al^3+^-induced cellular changes in arrangement, shape, size, maturity, inner structure and components would be important mechanisms for inhibiting IAA transport in root tips.

### Exogenous application of IAA alleviated Al^3+^-induced inhibition of plant growth by protecting cell structure in root tips

Growth reduction due to excess Al^3+^ is a common feature in most plant species^2^. In the present study, Al^3+^ caused a reduction in root length, root weight, root activity, and total chlorophyll content in leaves. This was in agreement with several previous studies^45^. The Al^3+^-induced inhibition of root growth and damage in physiology, however, was significantly alleviated by exogenous application of IAA. Accumulation of IAA in root tips is essential for root growth in conditions of excessive metal stress^46^,^47^. Exogenous application of auxin alleviated cadmium toxicity in *Arabidopsis* due to its compensation for the lack of auxin in the root tips^48^. Exogenous application of IAA protected the cell structures of Al^3+^-stressed root tips (cell arrangement, cell shape and cell size); consequently, it restored the convenient array of IAA fluorescence signals in the form of a dotted line and balanced the distribution of IAA fluorescence signals in different zones of the root tip. Those results suggested that the Al^3+^-induced inhibition of alfalfa growth could be alleviated by auxin through reducing the cell structure damage in root tips.

In conclusion, our results demonstrated that IAA played a key role in alfalfa responses to Al^3+^ stress. IAA synthesis and transport in alfalfa was sensitive to Al^3+^ stress, as exhibited by the quick decrease in IAA concentrations in apical buds, shoot base and root tips of alfalfa seedlings in response to Al^3+^ stress. The significant reduction of IAA accumulation in apical buds and root tips, as well as the imbalance of IAA distribution among the meristematic zone, transition zone and elongation zone of Al^3+^-stressed root tips may be the fundamental factors attributed to the restriction of plant growth by Al^3+^ stress. Al^3+^ stress caused serious damage to the cell structure in terms of cell arrangement, shape, size, maturity, inner structure and components, which strongly disturbed IAA transport in root tips. Moreover, exogenous application of IAA could partly alleviate this disturbance, restore the convenient array of IAA fluorescence signals, enhance IAA accumulation in root tips, and increase root growth.

## Material and Methods

### Plant materials and growth conditions

An Al^3+^-tolerant alfalfa genotype, WL-525HQ^49^, was obtained from the Chinese National Seed Group Corporation, Ltd. and was used in this study. Seeds were soaked in distilled water for 1 h and then germinated on a filter paper moistened regularly with ½-strength Hoagland’s nutrient solution in a growth chamber at a temperature of 25 °C. After six days, uniform seedlings were transplanted to a foam board (12 holes/plate; 6 seedlings/hole) floating on an aerated ½-strength Hoagland’s nutrient solution (pH 5.8) in plastic containers. The composition of Hoagland’s nutrient solution is 2.5 mM KNO_3_, 0.5 mM NH_4_NO_3_, 1 mM MgSO_4_, 0.5 mM KH_2_PO_4_, 2 mM Ca(NO_3_)_2_, 25 μM FeSO_4_, 25 μM Na_2_-EDTA, 2.5 μM KI, 50 μM MnSO_4_, 50 μM H_3_BO_3_, 0.05 μM CuSO_4_, 15 μM ZnSO_4_, 0.5 μM NaMoO_4_ and 0.05 μM CoCl_2_. Solutions were renewed every 2 d, and all seedlings were grown in growth chambers with a temperature of 25/20 °C (day/night), a 14-h photoperiod, photon flux density of 400 μmol m^−2^ s^−1^ and a relative humidity of 65%. Four days after transplanting (10 d after sowing), seedlings were used for the following treatments in the same growth chambers.

### Treatments and Experimental Design

For aluminium toxicity treatments, seedlings were treated with ½-strength Hoagland’s solution (pH 4.5) supplemented with 0 (-Al) or 100 μM AlCl_3_ (Al) (Supplementary Fig. 1) for 1, 3 and 10 d, and then plant samples were collected for further analysis. For root tip (1–10 mm) collection, both the main root tips and the secondary root tips were collected. For IAA treatments, plants exposed to 0 (-Al) or 100 μM AlCl_3_ (Al) were foliar sprayed with 2 ml of 6 mg L^−1^ IAA (Sigma, MO, USA) every two days, which comprised the -Al + IAA and Al + IAA treatments, and the control plants were sprayed with 2 ml water (pH 6.0). The 6 mg L^−1^ IAA concentration used for foliar spraying was selected from a preliminary experiment showing positive effects on mitigating Al^3+^ stress^50^. After 1, 3 and 10 d of IAA treatment, plant samples were collected for further analysis.

For microscopy study and immunohistochemical analysis of IAA in root tips, the alfalfa seeds were germinated in ½-strength Hoagland’s nutrient solution for 60 h and then supplemented with 0 (-Al) or 100 μM AlCl_3_ (Al) for 1, 3, and 10 days; the root tips (0–8 mm) were then cut for the microscopy study and immunohistochemical analysis.

In the apical buds removal experiment, to analyze the effect of Al^3+^ stress on IAA synthesis in apical buds, seedlings were divided into two groups: apical bud intact and apical bud removed. Apical buds of seedlings in the latter group were removed before being treated with aluminium and IAA. Each group was treated with aluminium and IAA using the method previously described for 24 h and was then sampled for IAA measurement.

To determine whether the deficiency of Zn^2+^ and Mg^2+^ in Al^3+^-stressed alfalfa influenced auxin synthesis, we designed the following treatments. After the first leaf of seedlings emerged under the growth condition of ½-strength Hoagland’s nutrient solution, roots of the seedlings were washed with deionized water and grown for 12 h in deionized water. Seedlings were then grown for 24 h in 0.5 mM Ca^2+^ solution (pH 4.5) with 0 (-Al) or 100 μM AlCl_3_ (Al). Among the Al^3+^ treatments, some of the plants received 25 μM or 50 μM MgCl_2_ for the Mg^2+^ treatment, and 25 μM or 50 μM ZnCl_2_ for the Zn^2+^ treatment.

### Physiological analysis

After 1, 3 and 10 d of treatment with aluminium and IAA, 10 plants from each container were separated into shoots and roots. The fresh weight of shoots was measured. Roots were blotted dry with paper towels and then weighed to determine the root fresh weight. Root length was measured from the root tip to the base of the main root. Membrane lipid peroxidation, expressed as malondialdehyde (MDA) content, was determined using the thiobarbituric acid (TBA) protocol^51^. Chlorophyll content was estimated according to the procedure described by Porra *et al*.^52^. Root activity was measured using the triphenyltetrazolium chloride (TTC) reduction method as described by Islam *et al*.^53^.

### Microscopy investigation

The collected root tips were immediately fixed in 2.5% (v/v) glutaraldehyde and 4% paraformaldehyde (Electron Microscopy Sciences) in PBS, pH 7.2, for 48 h at 4 °C. Samples were then washed with PBS, dehydrated in a graded ethanol series, and embedded in LR White resin (Ted Pella Inc.). Transverse and longitudinal sections (1 μm) were cut with a diamond knife on a Leica EM UC6 ultramicrotome (Leica Mikrosysteme). For Toluidine Blue O staining, 1-μm semi-thin sections were placed onto glass slides, stained with 1% (w/v) Toluidine Blue O (with 1% [w/v] sodium borate) for 5 min, and observed under a microscope (Olympus IX71, Olympus Optical Co. Ltd, Tokyo, Japan). The meristem length of the root tips was measured from the quiescent centre to the cortical cells, which are rapidly elongated, as described by Blilou *et al*.^41^. The meristem width was measured in the middle of the meristematic zone. The results presented are average values of more than 30 seedlings per treatment from three independent experiments.

### Immunohistochemical Analysis

Auxin immunolocalization was performed as described by Nishimura *et al*.^54^ and Watanabe *et al*.^55^. Excised root tips were immediately fixed in 4% 1-ethyl-3-(dimethylaminopropyl)-carbodiimide hydrochloride (EDAC) and 4% paraformaldehyde for 24 h at 4 °C. Samples were then dehydrated in a graded ethanol series, infiltrated with xylene, and embedded in paraffin wax using conventional methods. Material sections were cut to thicknesses of 10μm and pasted onto poly-lysine slides. The sections were then treated in a blocking solution consisting of 10 mM PBS/Tween-20/glycine/bovine serum albumin (BSA) (93.5/0.1/1.5/5, v/v/w/w) for 45 min, rinsed in regular-salt rinse solution (RSR) containing 10 mM PBS/Tween-20/BSA/NaCl (98/0.1/0.8/0.88, v/v/w/w) for 5 min, and treated with an additional solution containing 10 mM PBS/Triton X-100/BSA (99/0.8/0.1, v/v/w). Subsequently, the tissue sections were incubated in the primary rabbit polyclonal antibody to IAA (Abcam, UK) in the dark at 37^◦^C for 30 min. They were then washed in a high-salt rinse solution consisting of 10 mM PBS/Tween-20/BSA/NaCl (97/0.1/0.1/2.9, v/v/w/w). After several rinses in RSR and PBS, the tissue sections were incubated with the secondary antibody (anti-rabbit IgG Alexa Fluor 488-conjugate; Protein tech Group, Chicago, IL, USA) and were detected immune signals at 495 nm. Fluorescence was detected with a laser-scanning confocal microscope (Leica TCS SP5II).

Indole-3-acetic acid was extracted and purified according to the method described by Yan *et al*.^56^ with some modifications. A 0.5-g fresh sample was ground in liquid nitrogen, and then 5 ml pre-cooled (−20 °C) 80% methanol (containing 10 mg L^−1^ BHT, w/v) was added to the sample. After overnight extraction at −20 °C, solids were separated by centrifugation (20,000 g, 15 min) and re-extracted for 30 min in an additional 5 ml of the same extraction solution. Then, the supernatants were concentrated to 2.0 ml and were passed through a Sep-Pak Plus C18 cartridge (SepPak Plus, Waters, USA). After washing with 3 ml 20% methanol containing 1% (v/v) acetic acid, the cartridges were eluted with 1 ml pure methanol for HPLC measurement. The determination was repeated at least three times. Analyses were carried out on a Shimadzu LC-10A HPLC (Shimadzu, Kyoto, Japan) system equipped with an SPD-10Avp detector.

Callose content was measured according to the method described by Jones *et al*.^36^

### Gene expression analysis

Three genes related to auxin transport were examined with quantitative PCR (Q-PCR): auxin transporter-like protein (*AUX1*), and auxin efflux carrier components 1 and 2 (*PIN1* and *PIN2*). Total RNA was isolated using Trizol (Invitrogen). The first-strand cDNA was synthesized with a PrimeScript RT reagent kit (Takara) using oligo (dT) primers. For real-time RT-PCR analysis, 1 μl 10-fold-diluted cDNA was used for quantitative analysis of gene expression performed with SYBR Premix ExTaq (Takara) with the specific primers (Table 2).

Reactions were run on an ABI PRISM 5700 Sequence Detector (Applied Biosystems). Elongation factor 1-α (*EF1-α*) was selected as an endogenous control. Each gene was examined three times. All PCR efficiencies were above 95%. Sequence Detection Software (version 1.3, Applied Biosystems) results were exported as tab-delimited text files and imported into Microsoft Excel (Redmond, WA, USA) for further analysis. The median coefficient of variation (based on calculated quantities) of duplicated samples was 6%.

### Statistical analysis

The data were subjected to analysis of variance (ANOVA), and the least significant difference (LSD) test was employed to determine differences among the treatments at *P* = 0.05 levels.

## Additional Information

**How to cite this article**: Wang, S. *et al*. Aluminium-induced reduction of plant growth in alfalfa (*Medicago sativa*) is mediated by interrupting auxin transport and accumulation in roots. *Sci. Rep.* **6**, 30079; doi: 10.1038/srep30079 (2016).

## Supplementary Material

Supplementary Information

## Figures and Tables

**Table 1:** IAA concentrations in root tips, root base and shoot base, and IAA_[root tips]_/IAA_[shoot base]_ radio in alfalfa seedlings grown for 1, 3 and 10 days on ½-strength Hoagland’s medium (pH 4.5) with treatments of no Aluminum(-Al), Aluminum stress (Al) (100 μM AlCl_3_), Aluminum with additional IAA (Al + IAA) (100 μM AlCl_3_ + IAA) and -Al with additional IAA(-Al + IAA).

**Treatments**	**1d**				**3d**				**10d**			
	**Root tips**	**Root base**	**Shoot base**	Root tips/shoot base*	**Root tips**	**Root base**	**Shoot base**	Root tips/shoot base*	**Root tips**	**Root base**	**Shoot base**	Root tips/shoot base*
-Al	1.276 ± 0.215b	/	12.81 ± 1.784b	9.961	2.397 ± 0.407a	3.672 ± 0.481b	34.49 ± 1.230b	6.950	1.193 ± 0.131b	3.435 ± 0.420b	23.43 ± 0.504b	5.092
Al	0.185 ± 0.044c	/	11.93 ± 1.279b	1.551	0.219 ± 0.058b	6.079 ± 0.687a	17.79 ± 2.557c	1.231	0.315 ± 0.038c	5.117 ± 0.507a	13.53 ± 1.890d	2.328
Al + IAA	0.772 ± 0.189bc	/	22.75 ± 0.787a	3.393	0.348 ± 0.069b	5.491 ± 0.001a	42.83 ± 3.435a	0.813	0.562 ± 0.199c	6.084 ± 0.615a	19.10 ± 0.748c	2.942
-Al + IAA	2.506 ± 0.405a	/	24.13 ± 2.052a	10.385	2.996 ± 0.119a	2.941 ± 0.318b	43.82 ± 3.155a	6.837	1.721 ± 0.184a	4.905 ± 0.009ab	27.91 ± 0.491a	6.166

Data are means ± SE of three replicates. Columns with different letters indicate a significant difference at *P* < 0.05 (least significant difference test).

^*^Root tips/shoot base is the radio of 100·IAA_[root tips]_/IAA_[shoot base]_.

**Table 2:** Primer sequences used for Q-PCR.

**Gene**	**Forward sequence**	**Reverse sequence**	**GenBank accession**
Elongation factor 1-α (*EF1-α*)	5′-GCACCAGTGCTCGATTGC-3′	5′-TCGCCTGTCAATCTTGGTAACAA-3′	XM_003618727
Auxin transporter-like protein (*AUX1*)	5′-GACTACATACACTGCTTGGT-3′	5′-AGTGGCAGAAGGAATCGTTA-3′	XM_003623180
Auxin efflux carrier components 2 (*PIN2*)	5′-GATGCTGGTCTTGGAATGGC-3′	5′-ATTGCTATTGAGGTTGCCGC-3′	XM_003609978
Auxin efflux carrier components 1 (*PIN1*)	5′-GCTTCACCTGTCTCTGATGTG-3′	5′-CCACCTTTCTCACCTCCTTC-3′	XM_003619781
