# Supplementary material for: Aluminium-induced reduction of plant growth in alfalfa (Medicago sativa) is mediated by interrupting auxin transport and accumulation in roots
Source: Sci Rep. 2016 Jul 20;6:30079. doi: 10.1038/srep30079 (PMC4951802; doi:10.1038/srep30079)
Supplement: Supplementary Information [file srep30079-s1.pdf]

Aluminium-induced reduction of plant growth in alfalfa (*Medicago sativa*) is mediated by interrupting auxin transport and accumulation in roots

Shengyin Wang[1], Xiaoyan Ren[1], Bingru Huang[2], Ge Wang[3], Peng Zhou[1*], Yuan An[1, 4*]

[1] School of Biological and Agricultural Science, Shanghai Jiao Tong University, Shanghai 200240, P. R. China

[2] Department of Plant Biology and Pathology, Rutgers, The State University of New Jersey, New Jersey, NJ 08901, USA

[3] Instrumental Analysis centre of Shanghai Jiao Tong University, Shanghai Jiao Tong University, Shanghai 200240, P. R. China

[4] Key Laboratory of Urban Agriculture (South), Ministry of Agriculture, Shanghai 201101, China

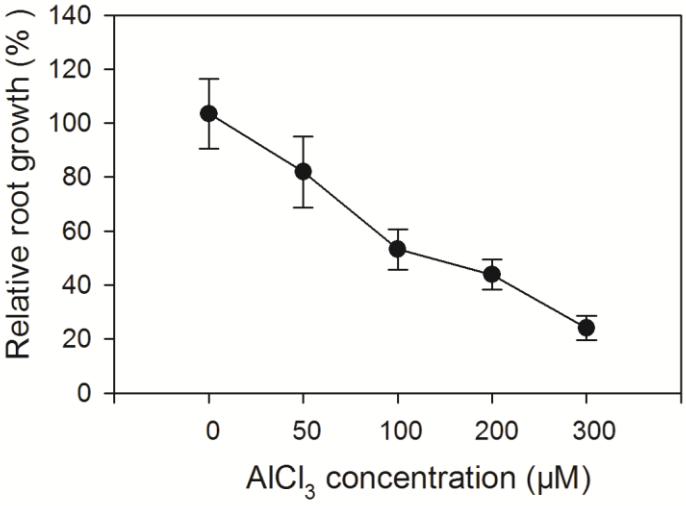

Supplementary Figure 1. The length of primary roots of alfalfa seedlings grown for 48h on ½-strength Hoagland's medium (pH 4.5) containing 0, 50, 100, 200 and 300 μM $AlCl_3$. Data are presented as relative root elongation compared with control values. Data are means ±SE of eight replicates. Bars with different letters indicate significant difference at $P < 0.05$ (least significant difference test).

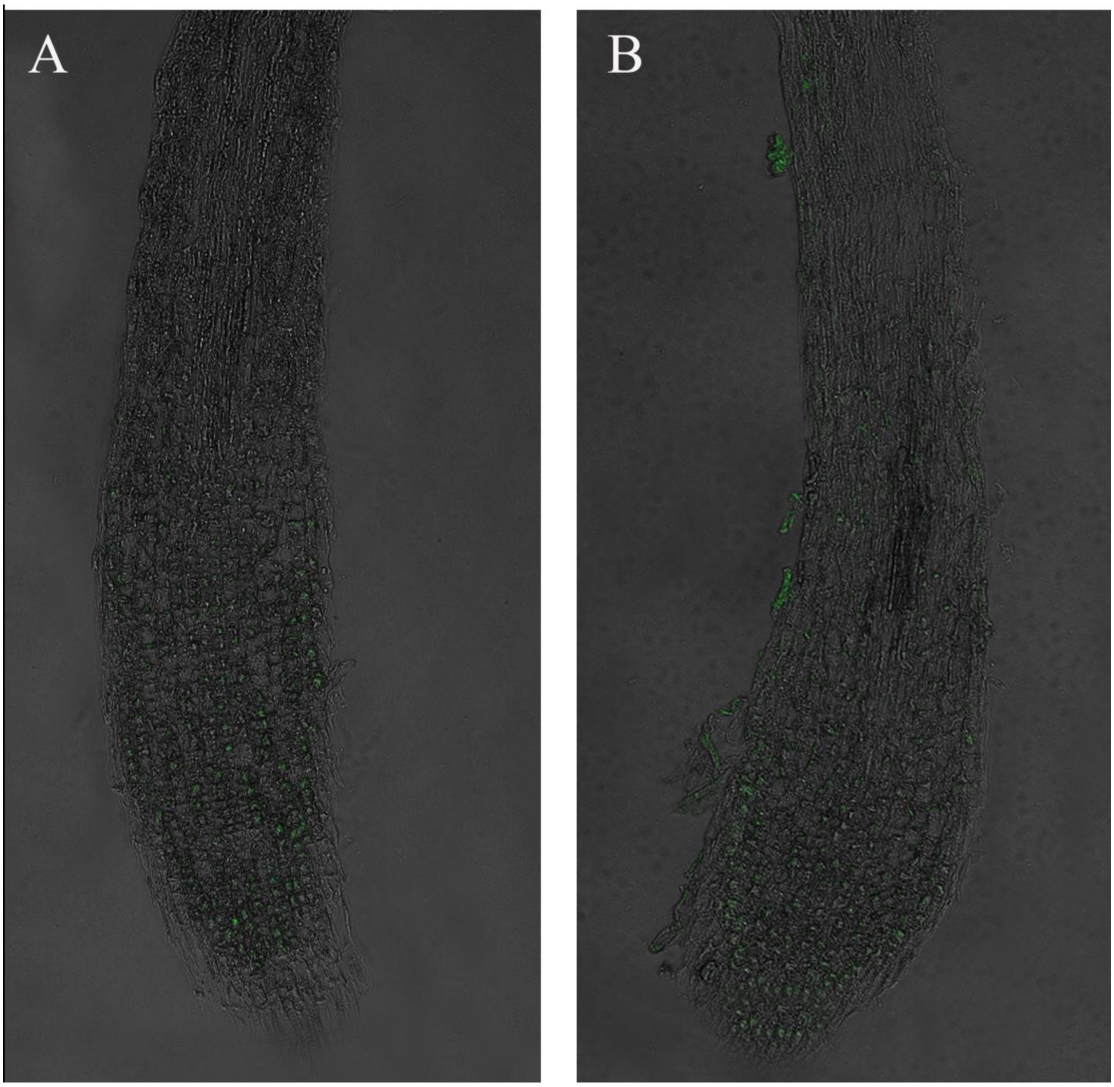

Supplementary Figure 2. Blank control (A) and negative control of IAA Immunolocalization in middle longitudinal section of alfalfa seedlings grown for 1d on ½-strength Hoagland's medium (pH 4.5) with treatments of no aluminum (-Al)(A), aluminum stress (Al)(B) (100 μM AlCl3). Scale bars in each image indicated 100 μm.
